# Morphological and molecular characterization of *Fasciola* isolates from livestock in Golestan province, northern Iran

**DOI:** 10.1002/vms3.1189

**Published:** 2023-06-15

**Authors:** Mitra Sharbatkhori, Saeid Nasibi, Mohammad Ali Mohammadi, Mojgan Aryaeipour, Saber Raeghi, Majid Fasihi Harandi

**Affiliations:** ^1^ Laboratory Sciences Research Center Golestan University of Medical Sciences Gorgan Iran; ^2^ Department of Medical Parasitology and Mycology School of Para‐Medicine Golestan University of Medical Sciences Gorgan Iran; ^3^ Research Center for Hydatid Disease in Iran Kerman University of Medical Sciences Kerman Iran; ^4^ Department of Medical Parasitology and Mycology School of Public Health, Tehran University of Medical Sciences Tehran Iran; ^5^ Department of Laboratory Sciences Urmia University of Medical Sciences Urmia Iran

**Keywords:** characterization, *Fasciola gigantica*, *Fasciola hepatica*, Iran, livestock, molecular analysis, morphometry, pepck, RFLP

## Abstract

**Background:**

Fascioliasis, caused by the liver flukes *Fasciola hepatica* and *Fasciola gigantica*, is a global zoonotic helminthic disease. The livestock and human are the final hosts of the parasites. Northern Iran is an important endemic region for fascioliasis. Few studies have been conducted on the characterization of *Fasciola* isolates from eastern regions of the Caspian littoral of the country.

**Objective:**

The aim of the present study was to identify *F. hepatica, F. gigantica* and intermediate/hybrid forms of *Fasciola* isolates from livestock in Golestan province, northern Iran, using morphometric and molecular tools.

**Methods:**

Livestock livers naturally infected with *Fasciola* spp. were collected from Golestan slaughterhouse during 2019–2020. The worms were morphometrically studied using a calibrated stereomicroscope. Genomic DNA was extracted from all samples, and polymerase chain reaction–restriction fragment length polymorphism (PCR–RFLP) was performed on internal transcribed spacer (ITS1) region using Rsa1 restriction enzyme. All the isolates were then analysed by multiplex PCR on Pepck region.

**Results:**

A total of 110 *Fasciola* isolates were collected from the infected livers, including 94 sheep, 12 cattle and 4 goats. Morphometric analysis of 61 adult *Fasciola* isolates indicated that, 44 and 17 isolates belonged to *F. hepatica* and *F. gigantica*, respectively. Eighty‐one and 29 isolates belonged to *F. hepatica* and *F. gigantica* using ITS1‐RFLP, respectively. However, Pepck Multiplex PCR indicated 72 *F. hepatica*, 26 *F. gigantica* and 12 intermediate/hybrid forms. All 12 hybrid isolates were found in sheep host. Two isolates were identified as *F. gigantica* using morphometry and *F. hepatica* using both molecular methods.

**Conclusion:**

The present study confirmed the existence of both *F. hepatica* and *F. gigantica* species and reported the first molecular evidence of hybrid *Fasciola* isolates in ruminants of Golestan province.

## Graphical abstract

The aim of the present study was to identify *Fasciola hepatica*, *Fasciola gigantica* and the intermediate/hybrid forms of *Fasciola* isolates from livestock in Golestan province, northern Iran, using morphometric and molecular tools. From a total of 110 Fasciola isolates collected from 94 sheep, 12 cattle and 4 goats, 72 isolates (65.4%) were identified as *F. hepatica*, 26 isolates (23.6%) as *F. gigantica* and 12 isolates (11.0%) as the hybrid forms. The present study confirmed the existence of both *F. hepatica* and *F. gigantica* species and reported the first molecular evidence of hybrid *Fasciola* isolates in ruminants of Golestan province.

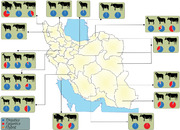

## 1 INTRODUCTION

*Fasciola hepatica* and *Fasciola gigantica* are the causative agents of fascioliasis, an important zoonotic disease that involve both human and livestock. *Fasciola* species cause significant economic losses due to liver rot, high morbidity rates and decreased milk, meat and wool production (Beesley et al., 2018; Mas‐Coma et al., 2019). Two large outbreaks of fasciolosis have been occurred in Gilan, the Northern province of Iran, in 1989 and 1999, with approximately 10,000 and 5,000 cases, respectively (Ashrafi et al., 2015). Both *Fasciola* species along with probable intermediate or hybrid forms are believed to present in northern Iran (Amor et al., 2011; Ashrafi et al., 2006).

Precise identification of *Fasciola* species is essential for understanding epidemiology, control strategies and management of fascioliasis in endemic areas, as different species of snail intermediate hosts are involved in the parasites’ life cycle with different ecological and biological features. Moreover, the hybrid forms can be observed where both species coexist (Mas‐Coma et al., 2009). Intermediate *Fasciola* isolates have been described from Asian countries (Amor et al., 2011), Vietnam (Itagaki et al., 2009; Le et al., 2008), China (Ichikawa‐Seki, Peng, et al., 2016; Peng et al., 2009), Japan (Itagaki et al., 2005), Korea (Agatsuma et al., 2000; Ichikawa & Itagaki, 2012) as well as from some regions of Africa in Egypt (Amer et al., 2011; Periago et al., 2008) and Chad (Evack et al., 2020).

Traditionally, the distinction between *Fasciola* species has been made based on morphological features. Adults of *F. hepatica* are shorter but wider, with longer cephalic cone, and a bigger shoulder, smaller ventral sucker and more exterior testes, compared to *F. gigantica* (Muller & Wakelin, 2002). Nevertheless, precise differentiation of the two flukes is often difficult because of some morphological variations among different isolates. DNA‐based molecular methods have been used to understand the nature and extent of inter‐ and intraspecific variations in *Fasciola*. Different tools and genetic markers have been used including polymerase chain reaction–restriction fragment length polymorphism (PCR–RFLP) on the nuclear internal transcribed spacers (ITS1 and/or ITS2) (Anh et al., 2018; Mir et al., 2019; Rokni et al., 2010; Saadatnia et al., 2022; Shafiei et al., 2013) and mitochondrial cytochrome c oxidase subunit 1 and/or NADH dehydrogenase 1 (Farjallah et al., 2013; Itagaki et al., 2005). However, using these molecular techniques, it is difficult to reliably differentiate intermediate or hybrid forms of *Fasciola*. Recent works have introduced new gene targets for differentiating the intermediate forms using multiplex‐PCR on pepck and PCR–RFLP of pold genes. This provided molecular tools for the precise identification of *Fasciola* species along with the hybrid forms in the endemic regions (Hayashi et al., 2018; Hayashi, Ichikawa‐Seki, et al., 2016; Tang et al., 2016). Unfortunately, sufficient data and information on the molecular identity of *Fasciola* isolates in this part of Caspian littoral is lacking. Therefore, to accurately differentiate *F. hepatica, F. gigantica* and intermediate/hybrid forms, this study conducted to characterize Iranian *Fasciola* isolates from northern Iran, using morphometry, ITS1‐RFLP and Pepck multiplex‐PCR.

## 2 MATERIALS AND METHODS

### 2.1 Parasite materials

A total of 110 *Fasciola* isolates were collected from the livers of infected sheep (94), cattle (12) and goats (4) during routine veterinary inspections between 2019 and 2020, in the central abattoir of Golestan province, northern Iran (N 37.111385, E 54.481278).

### 2.2 Morphometric study

For morphometric analysis, the worms were washed in PBS and fixed and tied between two slides. Morphology and morphometric characters of the flukes were studied using a microscope equipped with a drawing tube. The morphological parameters were recorded in an Excel spreadsheet. Morphometric analysis was performed using following criteria: body length (BL), body width (BW), body length to width ratio (BL/BW) and cephalic cone length.

Student's 𝑡‐test was employed for comparing the mean of different variables between *F. hepatica and F. gigantica*, and one‐way analysis of variance was used to find any significant differences between the means of morphometric values in flukes separated from different hosts (Sahba et al., 1972; Yamaguti, 1958). Thereafter, the flukes were fixed in 70% ethanol and stored at refrigerator until further molecular analysis.

### 2.3 ITS1 PCR–RFLP and Pepck Multiplex PCR

A small piece of lateral parts of each worm was removed and squashed inside a clean porcelain mortar containing 500 μL lysis buffer (0.1 M NaOH, 0.5 M Tris–HCl, 0.05 M EDTA and 1% SDS) using a pestle. Then the lysate transferred to a micro tube and incubated at 60°C for 3 h after adding 20 μL proteinase K. The lysate from each sample was subjected to DNA extraction using a conventional phenol–chloroform method with minor modifications (Sambrook et al., 1989). In the final step, the DNA was eluted in 50 μL deionized distilled water (DDW) and frozen at 20°C until the next step.

A piece of 680‐nucleotide fragment, including partial 18 s, complete ITS1 and partial 5.8 s, was amplified using the primers, ITS1‐F (5′‐TTGCGCTGATTACGTCCCTG‐3′) and ITS1‐R (5′‐TTGGCTGCGCTCTTCATCGAC‐3′) (Itagaki et al., 2005). Each 25 μL PCR reaction volume contained 9.5 μL DDW, 12.5 μL of PCR premix (2× Master Mix RED; Ampliqon, Odense, Denmark), 1 μL of each ITS1 primer (10 pmol/μL) (Arian Gene Gostar, Tehran, Iran) and 1 μL DNA template. The reaction mixture was amplified in a thermocycler (PeqStar, Germany) under the following conditions: 94°C for 5 min as initial denaturation followed by 30 cycles at 94°C for 2 min as denaturation, 59°C for 30 s as annealing, 72°C for 45 s as extension and a final extension at 72°C for 10 min. In each set of PCR, DDW was used instead of DNA template as negative control.

Five microliters of PCR products were analysed by electrophoresis in SYBR green‐stained 1.5% agarose gels prepared in TBE buffer (89 mM Tris, 89 mM boric acid and 2 mM EDTA, pH 8.3) at 80 V for 1 h. A 100‐bp DNA ladder (Sinaclon, Tehran, Iran) was run to show the size of DNA fragments in each gel. Gels were visualized and photographed on a transilluminator (Qiagen, Germany).

Ten microliters of each ITS1‐PCR product were digested for 4 h at 37°C by Rsa1 restriction endonucleases (Thermo Scientific, Waltham, USA), in a final volume of 30 μL according to the manufacturer instructions. Restriction fragments along with a 50‐bp DNA ladder (Sinaclon, Tehran, Iran) were separated by electrophoresis through 2.5% agarose gels, stained with SYBR green, and visualized by UV transilluminator.

To amplify partial PEPCK gene, a multiplex PCR was employed using the following primers: Fh‐pepck‐F (5′‐GATTGCACCGTTAGGTTAGC‐3′), Fg‐pepck‐F (5′‐AAAGTTTCTATCCCGAACGAAG‐3′) and Fcmn‐pepck‐R (5′‐CGAAAATTATGGCATCAATGGG‐3′) (Hayashi et al., 2018). Multiplex PCR reactions (25 μL) contained 14 μL DDW, 6 μL of PCR premix (2× Master Mix RED; Ampliqon, Odense, Denmark), 1 μL of each primer (10 pmol/μL) (Metabion, Munich, Germany) and 2 μL DNA template.

The multiplex PCR amplification was performed in a thermocycler (Flex cycler, Germany) under the following temperature conditions: 94°C for 5 min as initial denaturation followed by 30 cycles at 94°C for 30 s as denaturation, 59°C for 30 s as annealing, 72°C for 1 min as extension and a final extension at 72°C for 10 min. In each set of multiplex‐PCR, DDW was used for negative control. Two known *F. hepatica* and *F. gigantica* isolates were used as positive controls. Five microliters of multiplex PCR products were analysed by electrophoresis in SYBR green‐stained 2% agarose gels prepared in TBE buffer (89 mM Tris, 89 mM boric acid and 2 mM EDTA, pH 8.3) at 80 V for 40 min. A 100‐bp DNA ladder (Paras Tools, Mashhad, Iran) was run to show the size of amplicon in each gel. DNA fragments were visualized and photographed on a transilluminator (Uvitec, UK).

## 3 RESULTS

### 3.1 Morphometric analysis

Of 110 *Fasciola* isolates, morphometric analysis was performed on 61 specimens as some of the helminths were juvenile worms without full‐grown reproductive organs. The morphometric characteristics of isolated flukes are summarized in Table 1. The mean length of *F. gigantica* (2.5–4.76 cm) was greater than *F. hepatica* (1.8–3 cm). The differences between the BL, CL, in the two species were significant (*p* < 0.05). Based on BL/BW and BW/CW ratios, 44 and 17 specimens were morphologically identified as *F. hepatica* and *F. gigantica*, respectively. No hybrid forms were identified by morphometric analysis. The measure of BL/BW has been considered one of the useful indices for differentiating *F. hepatica* from *F. gigantica*. The BL/BW ratios of 1.29–2.80 and 3.40–6.78 are considered for *F. hepatica* and *F. gigantica*, respectively (Periago et al., 2006). Forty‐four and 17 adult specimens had the BL/BW values in the range of those reported for *F. hepatica* and *F. gigantica*, respectively. The BL/BW ratios of 1.58–2.9 and 3.2–6.6, the (BW/CW) ratios of 2.1–5.55 and 1.5–4 and (VS/OS) ratios of 0.3–2.5 and 1–3.8 are considered for *F. hepatica* and *F. gigantica*.

**TABLE 1:** Comparative morphological data of adult *Fasciola* isolates from naturally infected livestock in Golestan province, northern Iran.

Morphometric character	*F. hepatica* range (cm) (Mean ± SD)	*F. gigantica* range (cm) (Mean ± SD)
**Body length (BL)**	1.8–3 (2.4 ± 0.32)	2.5–4.76 (3.33 ± 0.59)
**Body width (BW)**	0.71–1.5 (1.17 ± 0.17)	0.5–1.13 (0.8 ± 0.18)
**Cone length (CL)**	0.12–0.33 (0.21 ± 0.05)	0.17–0.33 (0.22 ± 0.04)
**Cone width (CW)**	0.24–0.45 (0.32 ± 0.04)	0.3–0.45 (0.34 ± 0.04)
**Oral sucker (OS)**	0.03–0.11 (0.06 ± 0.02)	0.04–0.1 (0.07 ± 0.02)
**Ventral sucker (VS)**	0.07–0.14 (0.11 ± 0.02)	0.04–0.3 (0.14 ± 0.06)
**Distance between suckers (OS–VS)**	0.06–0.22 (0.13 ± 0.03)	0.04–0.21 (0.11 ± 0.04)
**Testicular space length (TL)**	0.68–1.6 (1.18 ± 0.24)	0.76–1.97 (1.36 ± 0.28)
**Testicular space width (TW)**	0.39–1.1 (0.67 ± 0.14)	0.34–0.76 (0.47 ± 0.1)
**BL/BW**	1.58–2.9 (2.08 ± 0.3)	3.2–6.6 (4.29 ± 1.04)
**BW/CW**	3–5.55 (3.63 ± 0.59)	1.5–3 (2.39 ± 0.51)
**CW/CL**	0.73–3.2 (1.71 ± 0.45)	1.33–1.83 (1.54 ± 0.18)
**VS/OS**	0.3–2.5 (1.78 ± 0.44)	1–3.8 (2 ± 0.72)

### 3.2 Molecular analysis

**FIGURE 1:**
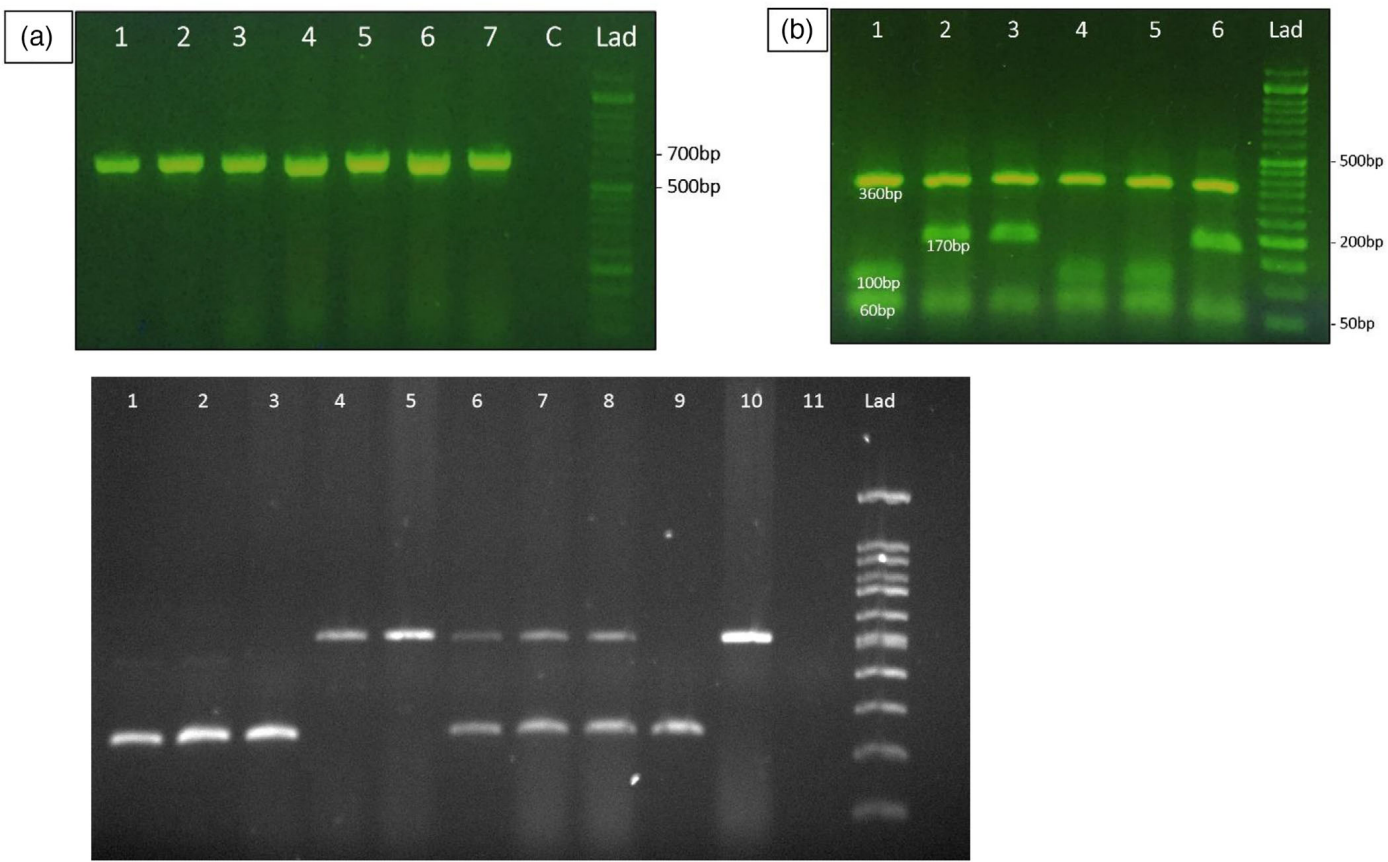
Agarose gel electrophoresis of the amplicons produced from different *Fasciola* gene targets: (a) 680‐bp fragment produced from internal transcribed spacer‐polymerase chain reaction (ITS1‐PCR); (b) restriction fragment length polymorphism (RFLP) profile obtained after using Rsa1 restriction enzyme on rDNA ITS1 region, revealing three fragments of 60, 100 and 360 bp for *F. hepatica* and 60, 170 and 360 bp for *Fasciola gigantica* isolates. (c) Multiplex PCR of PEPCK gene of *Fasciola* flukes isolated from livestock in Golestan province. 1–3: *Fasciola hepatica* flukes from sheep, cattle and goat (isolates ID 209, 234 and 238); 4–5: *F. gigantica* flukes from sheep and cattle (112, 118 isolates); 6–7 *Fasciola* hybrid forms (isolates ID 230,42); 8: *Fasciola* hybrid control; 9: *F. hepatica* control; 10: *F. gigantica* control; 11: Blank control; Lad: DNA size marker.

In ITS1‐PCR a 680 bp fragment was visualized after gel electrophoresis (Figure 1a). ITS1‐RFLP using Rsa1 restriction enzyme revealed three fragments of 60, 100 and 360 bp for *F. hepatica* and 60, 170 and 360 bp for *F. gigantica* isolates (Figure 1b). Using ITS1‐PCR–RFLP, out of 110 *Fasciola* isolates, 81 (73.6%) and 29 (26.4%) isolates were identified as *F. hepatica* and *F. gigantica*, respectively. Pepck‐Multiplex PCR amplified a 241 bp fragment for *F. hepatica*, 510 bp for *F. gigantica*. The hybrid forms demonstrated both fragments in gel electrophoresis (Figure 1c). Using Pepck‐Multiplex PCR, 72 isolates (65.4%) were identified as *F. hepatica* and 26 isolates (23.6%) as *F. gigantica*, whereas 12 isolates (11.0%) were identified as hybrid forms. All 12 hybrid *Fasciola* specimens were isolated from sheep host. All four goat isolates were identified as *F. hepatica* by morphometry as well as the both molecular methods. Overall, 63 (67%), 19 (20.2%) and 12 (12.8%) of 94 sheep isolates were belonged to *F. hepatica, F. gigantica* and hybrid forms, respectively. Five (41.66%) and 7 (57.33%) cattle isolates belonged to *F. hepatica* and *F. gigantica*, respectively. Two sheep isolates, first identified as *F. gigantica* using morphometry, were characterized as *F. hepatica* by the both molecular methods. Moreover, a sheep isolate identified as *F. hepatica* using ITS1‐RFLP was characterized as *F. gigantica* using morphometry and Pepck multiplex‐PCR. Another sheep isolate, indicated as *F. gigantica* using ITS1‐RFLP, was found to be *F. hepatica* by using morphometry and Pepck multiplex‐PCR.

**FIGURE 2:**
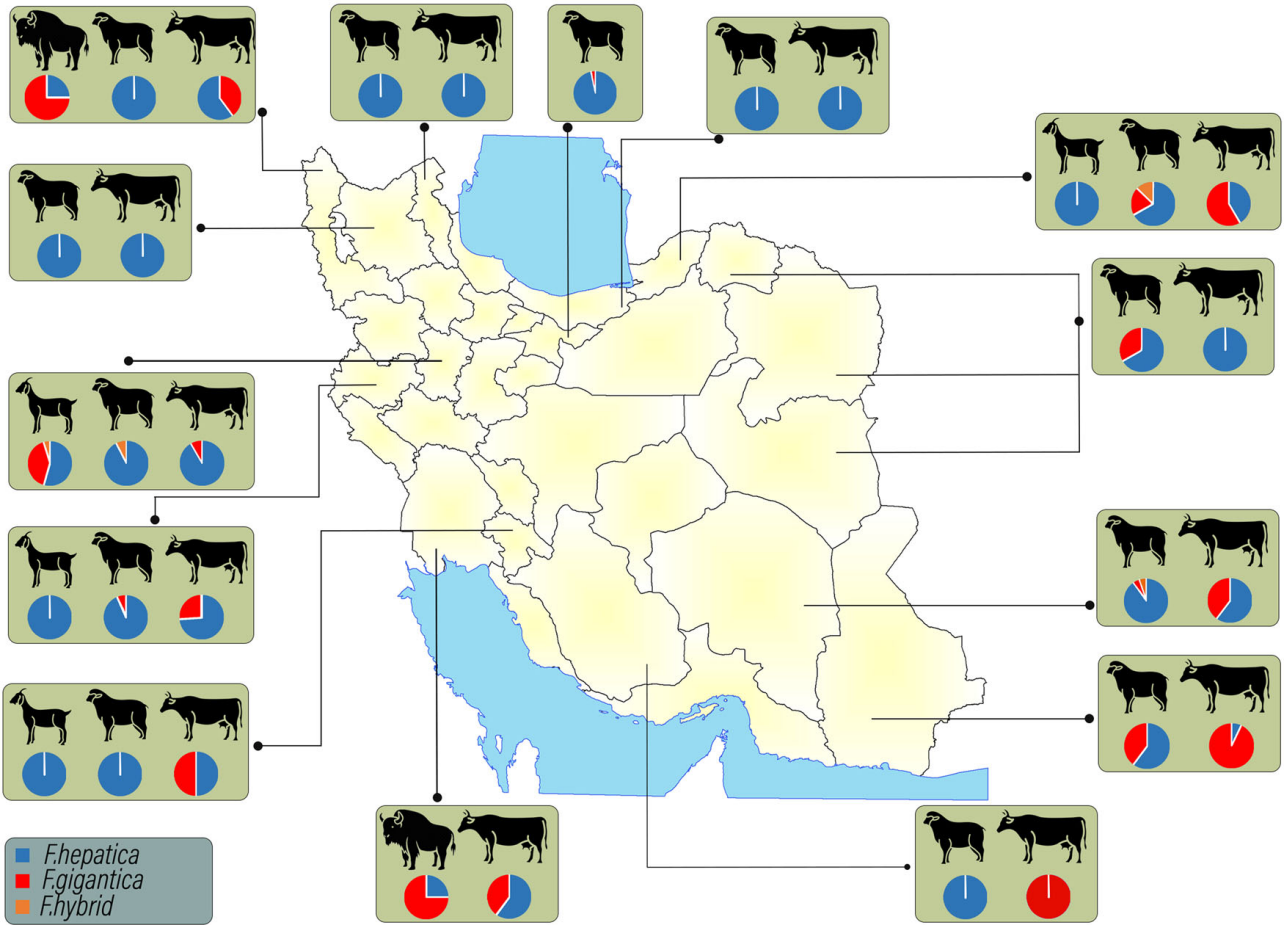
Host‐based geographical distribution of *Fasciola hepatica*, *Fasciola gigantica* and the hybrid forms in different livestock species in Iran (see Table 2 for the details and references).

## 4 DISCUSSION

Traditionally, identification of *F. hepatica* and *F. gigantica*, the main *Fasciola* species infecting human and animals, has been performed using morphologic and morphometric methods. However, the two species cannot be accurately discriminated by these methods. In addition, during past decade, the intermediate forms of *Fasciola* have been described from some Asian and African countries where both *F. hepatica* and *F. gigantica* coexist. Hybrid *Fasciola* flukes are believed to be originated from the interspecific hybridizations occurring throughout East, South‐east and South Asian countries. However, to our knowledge, the distribution of hybrid *Fasciola* flukes in the regions between Central Asia and the Middle East has not been investigated. Several previous reports attempted to identify the species of *Fasciola* flukes from these regions using morphological and morphometric features as well as conventional molecular markers (nuclear internal transcribed spacer 1 and 2) (Anh et al., 2018; Ichikawa & Itagaki, 2010; Ichikawa‐Seki, Ortiz, et al., 2016; Mizani et al., 2015; Teofanova et al., 2011); however, these findings have not been validated using more reliable nuclear gene markers. Recently, the nuclear genes, phosphoenol pyruvate carboxykinase (pepck) and DNA polymerase delta (pold), were found to be useful novel markers for the precise discrimination of *F. hepatica, F. gigantica* and hybrid *Fasciola* flukes without the need for gene sequencing that is expensive and not cost‐effective, particularly in the endemic countries (Hayashi et al., 2018; Hayashi, Ichikawa‐Seki, et al., 2016; Kasahara et al., 2021; Tang et al., 2016; Thang et al., 2019).

*F. hepatica* is more frequent than *F. gigantica* in endemic regions of the world (Mucheka et al., 2015; Tolan, 2011). However, the results of a study support that *F. gigantica* might be the main causal agent of fascioliasis in some northern regions of the Iran (Ashrafi et al., 2004). Moreover, specimens of *Fasciola* sp are reported as ‘intermediate forms’ (Le et al., 2008; Rehman et al., 2021; Sumruayphol et al., 2020). The life cycle of *Fasciola* spp. involves a very wide range of domestic and wild mammals, including sheep, cattle, horse, camel, coypu, rabbit as well as humans as definitive hosts (Akhlaghi et al., 2018; Beesley et al., 2018; Mas‐Coma et al., 2020; Sabourin et al., 2018). *Galba truncatula* is the main intermediate host in the old world. The snail intermediate hosts of fascioliasis in Iran proved to be *G. truncatula* and *Radix auricularia* (*Lymnaea gedrosiana*) (Eliazian & Akbarzadeh, 1979) and the role of *Stagnicola palustris* in transmission is doubtful (Aryaeipour et al., 2022; Salahi‐Moghaddam & Arfaa, 2013).

In the present study, among 61 adult worm isolates, morphometric analysis indicated the presence of *F. hepatica* (72.13%) and *F. gigantica* (27.87%). ITS1‐PCR–RFLP showed that 73.6% and 26.4% of 110 young and adult *Fasciola* isolates belonged to *F. hepatica* and *F. gigantica*, respectively, with no hybrid form. However, Pepck‐Multiplex PCR reveled 65.5%, 23.6% and 10.9% of 110 isolates as *F. hepatica, F. gigantica* and *Fasciola* hybrid form, respectively. It confirms that morphometric and PCR–RFLP analyses cannot accurately detect *Fasciola* species. Previous study in Golestan province using morphological method indicated 11.84% of sheep and cattle isolates belonged to *Fasciola* sp.; however, this was not confirmed by molecular methods (Halakou et al., 2017). We found two sheep isolates identified as *F. gigantica* using morphometry and as *F. hepatica* using both molecular methods. Moreover, we observed discrepancy among results of other two sheep isolates using three methods. It is recommended to follow a multi‐locus approach for molecular characterization of helminth parasites of human and veterinary importance.

Historically, *F. hepatica* is more common in temperate regions, including Europe, the Americas and Oceania, whereas *F. gigantica* is environmentally adapted to the tropical and humid regions such as Africa and Asia (Mas‐Coma et al., 2009). As shown in Figure 2, some studies in Iran have identified *F. hepatica* as the only species in sheep, goats and cattle. In Iran, some studies have identified *F. hepatica* as the only species in sheep, goats and cattle. In a study from 3 provinces in western Iran, all isolates, including 20 sheep, 29 cattle, 4 goats and 1 human isolate, belonged to F. hepatica species (Javanmard et al., 2020). Another study in the eastern provinces of Khorasan Razavi and North Khorasan indicated all 90 cattle isolates belonged to *F. hepatica* species (Raeghi et al., 2016). A study from East Azerbaijan province, north‐western Iran, reported all 72 stool isolates from sheep and cattle as the *F. hepatica* (Baran et al., 2017). In West Azerbaijan, north‐western Iran, all 40 sheep and 50 cattle isolates were *F. hepatica* (Galavani et al., 2016). There are similar results in other countries. For example, all the 225 *Fasciola* isolates from 7 sheep, 73 cattle and 1 pig from 18 distinct geographic areas in Ecuador, South America, identified as *F. hepatica* (Kasahara et al., 2021).

There are some other studies in Iran that reported *F. hepatica* as the more prevalent species in all hosts (Table 2). In Tabriz city, north‐western Iran, among 50 sheep and 50 cattle isolates, 75% and 25% belonged to *F. hepatica* and *F. gigantica*, respectively (Shahbazi et al., 2010). In western province of Kermanshah 94%, 96% and 100% of sheep, cattle and goat isolates were *F. hepatica*, respectively, western Iran (Bozorgomid et al., 2020). In a study from Hamadan province, western Iran, 92.7% of sheep, 54.5% of goats and 91.7% of cattle isolates belonged to *F. hepatica* (Piri et al., 2018). The present study, also, indicated the *F. hepatica* as the dominant species (65.5%) among 110 isolates. All four goat isolates in the present study were *F. hepatica*, which is in concordance with previous studies in Iran (Bozorgomid et al., 2020; Javanmard et al., 2020; M. Rokni et al., 2020; Shafiei et al., 2014).

**TABLE 2:** Summary of the molecular studies on *Fasciola* species infecting different species of livestock in different provinces of Iran.

Province	Host (*n*)	Method	No. *F. hepatica* (%)	No. *F. gigantica* (%)	No. Hybrid forms (%)	Reference
**Tehran, Khuzestan and West Azerbaijan**	Sheep (31)	ITS1‐RFLP (Tas1)	30 (96.8)	1 (3.2)		Rokni et al. (2010)
	Cattle (20)		12 (60)	8 (40)		
	Buffalo (28)		7 (25)	21 (75)		
	Goat (1)		1 (100)			
**Khorasan**	Sheep (15)	ITS1+5.8s+ITS2 RFLP (Tsp509I)	10 (66.7)	5 (33.3)		Mahami‐Oskouei et al. (2011)
	Cattle (15)		15 (100)			
**East Azerbaijan**	Sheep (15)		15 (100)			
	Cattle (15)		15 (100)			
**Fars**	Sheep (15)		15 (100)			
	Cattle (15)			15 (100)		
**East Azerbaijan**	Sheep (50) and cattle (50)	ITS1‐RFLP (Tas1)	75 (75)	25 (25)		Shahbazi et al. (2010)
**Mazandaran**	Buffalo (14) and goat (8)	ITS1 and ITS2 sequencing	25 (48.1)	20 (38.45)	7 (13.45)	Amor et al. (2011)
**Ardabil**	Sheep (35)	18s+ITS1‐RFLP (Rsa1)	35 (100%)			Aryaeipour et al. (2014)
	Cattle (35)		31 (88.6)	4 (11.4)		
**Kohkiluye and Boyer Ahmad**	Sheep (13)	ITS1‐RFLP (Rsa1, Msp1 and Kpn1)	13 (100)			Shafiei et al. (2014)
	Cattle (34)		17 (50)	17 (50)		
	Goat (11)		11 (100)			
**West Azerbaijan**	Sheep (40)	ITS1+5.8s+ITS2 sequencing	40 (100)			Galavani et al. (2016)
	Cattle (50)		50 (100)			
**Northern Khorasan, Razavi Khorasan**	Cattle (30)	ITS1‐RFLP (Rsa1), cox1 and nad1 sequencing	90 (100)			Raeghi et al. (2016)
**Kerman, Alborz**	Sheep (44)	ITS1, ITS2 and cox1 sequencing	42 (95.5)		2 (4.5)	Amor et al. (2011)
	Cattle (10)		6 (60)	4 (40)		
**Hamadan**	Sheep (41)	ITS1‐RFLP (Rsa1)	38 (92.7)		3 (7.3)	Piri et al. (2018)
	Cattle (12)		11 (91.7)	1 (8.3)		
	Goat (22)		12 (54.5)	9 (41)	1 (4.5)	
**Sistan and Baluchestan**	Sheep (43)	ITS1‐RFLP (Rsa1)	43 (100)			Mirahmadi et al. (2018)
	Cattle (50)		3 (6)	47 (94)		
**Sistan and Baluchestan**	Sheep (35)	ITS1‐RFLP (Rsa1)	4 (11.4)	31 (88.6)		Mir et al. (2019)
	Cattle (35)		3 (8.5)	32 (91.5)		
**Kermanshah**	Sheep (16)	ITS1‐RFLP (Rsa1)	15 (94)	1 (6)		Bozorgomid et al. (2020)
	Cattle (28)		27 (96)	1 (4)		
	Goat (4)		4 (100)			
**Kermanshah**	Sheep (16)	ITS1‐RFLP (Rsa1)	15 (93.75)	1 (6.25)		Rokni et al. (2020)
	Cattle (48)		29 (60.4)	19 (39.6)		
	Goat (4)		4 (100)			
**Ilam, Lorestan and Khuzestan**	Sheep (20)	ITS1, cox1, nad1 sequencing, Pepck multiplex PCR and pold RFLP (Alu1)	20 (100)			Javanmard et al. (2020)
	Cattle (29)		29 (100)			
	Goat (4)		4 (100)			
**Golestan**	Sheep (94)	ITS1‐RFLP (Rsa1), Pepck multiplex PCR	63 (67)	19 (20.2)	12 (12.8)	This study
	Cattle (12)		5 (41.7)	7 (58.3)		
	Goat (4)		4 (100)			

However, there are a few studies in Iran reported *F. gigantica* as the more prevalent species in livestock (Figure 2). In a study in Zabol, a city of Sistan and Baluchestan province, south‐eastern Iran, 88.6% and 91.5% of sheep and cattle isolates, belonged to *F. gigantica*, respectively (Mir et al., 2019). Another study in Ardabil province, north‐western Iran, reported *F. gigantica* in 88.6% of cattle isolates, whereas 100% of sheep isolates were *F. hepatica* (Aryaeipour et al., 2014).

In some countries, *F. gigantica* is the dominant species in livestock. For example, in Delhi, India, all the 40 *Fasciola* isolates from 11 buffalos belonged to *F. gigantica* (Hayashi, Mohanta, et al., 2016). Moreover, 96.9% of *Fasciola* isolates from cattle and buffalo in Nghe An province, Vietnam, belonged to *F. gigantica* (Anh et al., 2018).

The hybrid form may occur in some endemic areas where both *Fasciola* species coexist. The intermediate/hybrid forms of *Fasciola* have been mostly reported from Asian countries, such as Vietnam (Itagaki et al., 2009; Le et al., 2008), China (Ichikawa‐Seki, Peng, et al., 2016; Peng et al., 2009), Japan (Itagaki et al., 2005) and Korea (Choe et al., 2011). There are a few reports of this form in Iran. Ashrafi et al. (2006) described the intermediate form of *Fasciola* in Gilan province, northern Iran, using morphometric characterization. Amor et al. (2011) using ITS1 and ITS2 sequences reported seven intermediate forms of *Fasciola* (13.45%) from buffalo and goat in Mazandaran province, northern Iran. Akhlaghi et al. (2018) reported 2 out of 44 sheep isolates as *Fasciola* sp., employing cox1 and ITS1 sequencing. Phylogenetic analysis indicated that the two *Fasciola* sp. isolates were located in a same clade with an intermediate form of *Fasciola* sp. from Japan. The present study also reports *Fasciola* intermediate/hybrid form in 12 sheep isolates from Golestan province using Pepck‐Multiplex PCR.

## 5 CONCLUSION

This study confirmed the presence of *F. hepatica, F. gigantica* and the intermediate/hybrid forms in sheep, goat and cattle in the northern province of Golestan. Findings of the present study indicate the coexistence of both species of *Fasciola* and a high potential of fascioliasis transmission in the livestock of the region. The study presents the first molecular evidence of intermediate/hybrid forms of *Fasciola* from the sheep in this province.

## AUTHOR CONTRIBUTIONS

*Conceptualization; funding acquisition; methodology; project administration; resources; supervision; validation; writing – original draft; writing – review and editing*: Mitra Sharbatkhori. *Data curation; investigation; methodology; software; validation; visualization; writing – original draft; writing – review and editing*: Saeid Nasibi. *Formal analysis; methodology; software; writing – original draft; writing – review and editing*: Mohammad Ali Mohammadi. *Data curation; investigation; methodology; project administration; writing – original draft; writing – review and editing*: Mojgan Aryaeipour. *Investigation; methodology; validation; writing – original draft; writing – review and editing*: Saber Raeghi. *Conceptualization; formal analysis; methodology; project administration; validation; visualization; writing – original draft*: Majid Fasihi Harandi.

## CONFLICT OF INTEREST STATEMENT

The authors declare no conflict of interest.

## FUNDING INFORMATION

Golestan University of Medical Sciences, Gorgan, Iran, Grant Number: 17305.

### PEER REVIEW

The peer review history for this article is available at https://publons.com/publon/10.1002/vms3.1189.

## Data Availability

The raw data supporting the findings of the study are available upon reasonable request.
